# Tailored implementation of internet-based cognitive behavioural therapy in the multinational context of the ImpleMentAll project: a study protocol for a stepped wedge cluster randomized trial

**DOI:** 10.1186/s13063-020-04686-4

**Published:** 2020-10-28

**Authors:** Leah Bührmann, Josien Schuurmans, Jeroen Ruwaard, Margot Fleuren, Anne Etzelmüller, Jordi Piera-Jiménez, Tracy Finch, Tim Rapley, Sebastian Potthoff, Bruno Aouizerate, Philip J. Batterham, Alison Calear, Helen Christensen, Claus Duedal Pedersen, David Daniel Ebert, Erik Van der Eycken, Naim Fanaj, Claire van Genugten, Denise Hanssen, Ulrich Hegerl, Juliane Hug, Annet Kleiboer, Kim Mathiasen, Carl May, Sevim Mustafa, Caroline Oehler, Arlinda Cerga-Pashoja, Catherine Pope, Gentiana Qirjako, Judith Rosmalen, Ylenia Sacco, Ludovic Samalin, Mette Maria Skjøth, Kristine Tarp, Ingrid Titzler, Enrico Zanalda, Isabel Zbukvic, Johannes H. Smit, Heleen Riper, Christiaan Vis, on behalf of the ImpleMentAll consortium, Alison Calear, Andia Meksi, Anna Sofie Rømer, Anne Etzelmüller, Antoine Yrondi, Arlinda Cerga-Pashoja, Bridianne O’Dea, Bruno Aouizerate, Carl May, Carmen Ceinos, Caroline Oehler, Catherine Pope, Christiaan Vis, Claire van Genugten, Claus Duedal Pedersen, Corinna Gumbmann, David Daniel Ebert, Denise Hanssen, Els Dozeman, Enrico Zanalda, Erik Van der Eycken, Eva Fris, Gentiana Qirjako, Géraldine Visentin, Heleen Riper, Helen Christensen, Ingrid Titzler, Isabel Zbukvic, Jeroen Ruwaard, Johanna Freund, Johannes H. Smit, Jordi Piera-Jiménez, Josep Penya, Josien Schuurmans, Judith Rosmalen, Juliane Hug, Kim Mathiasen, Kristian Kidholm, Kristine Tarp, Leah Bührmann, Ludovic Samalin, Maite Arrillaga, Margot Fleuren, Marion Leboyeer, Martine Pool, Mette Atipei Craggs, Mette Maria Skjøth, Naim Fanaj, Philip J. Batterham, Pia Driessen, Robin Kok, Sebastian Potthoff, Sergi García Redondo, Sevim Mustafa, Tim Rapley, Tracy Finch, Ulrich Hegerl, Ylenia Sacco

**Affiliations:** 1 Department of Clinical, Neuro-, & Developmental Psychology, Faculty of Behavioural and Movement Sciences, VU Amsterdam, Amsterdam, The Netherlands; 2 Amsterdam UMC, Vrije Universiteit Amsterdam, Psychiatry, Amsterdam Public Health Research Institute, Amsterdam, The Netherlands; 3 Department of Research and Innovation, GGZ inGeest Specialized Mental Health Care, Amsterdam, The Netherlands; 4 Dutch Nurses Association, Utrecht, The Netherlands; 5 GET.ON Institute, Hamburg, Germany; 6 Department of Research and Innovation, Badalona Serveis Assistencials, Badalona, Spain; 7 Department of Nursing, Midwifery and Health, Northumbria University, Newcastle upon Tyne, UK; 8 Department of Social Work, Education and Community Wellbeing, Northumbria University, Newcastle upon Tyne, UK; 9 Faculty of Health and Life Sciences, Northumbria University, Newcastle upon Tyne, UK; 10 Fondation FondaMental, Creteil, France; 11 Regional Reference Center for the Management and Treatment of Anxiety and Depressive Disorders, Expert Center for Treatment-Resistant Depression, CH Charles Perrens, University of Bordeaux, Bordeaux, France; 12 Centre for Mental Health Research, Research School of Population Health, The Australian National University, Canberra, Australia; 13 Black Dog Institute, University of New South Wales, Randwick, Australia; 14 Centre for Innovative Medical Technology, Odense University Hospital, Odense, Denmark; 15 Department of Clinical Psychology and Psychotherapy, Friedrich-Alexander University of Erlangen-Nuremberg, Erlangen, Germany; 16 GAMIAN Europe, Brussels, Belgium; 17 Mental Health Center Prizren, Prizren, Kosovo; 18 College of Medical Sciences Rezonanca, Prishtina, Kosovo; 19 University Medical Center Groningen, Interdisciplinary Center for Psychopathology and Emotion Regulation, University of Groningen, Groningen, The Netherlands; 20 Department of Psychiatry, Psychosomatics and Psychotherapy, Goethe-Universität Frankfurt, Frankfurt, Germany; 21 European Alliance Against Depression e.V., Leipzig, Germany; 22 Research Unit for Depression and Anxiety, Aarhus University Hospital, Aarhus N, Denmark; 23 Faculty of Public Health and Policy, London School of Hygiene and Tropical Medicine, London, UK; 24 Faculty of Education, University St. Kliment Ohridski, Bitola, North Macedonia; 25 Stiftung Deutsche Depressionshilfe, Leipzig, Germany; 26 Department of Population Health, London School of Hygiene & Tropical Medicine, London, UK; 27 Nuffield Department of Primary Care Health Sciences, University of Oxford, Oxford, UK; 28 Department of Public Health, Faculty of Medicine, University of Medicine, Tirana, Albania; 29 Dipartimento di Salute Mentale, Azienda Sanitaria Locale Torino 3, Turin, Italy; 30 Department of Psychiatry, CHU Clermont-Ferrand, University of Clermont Auvergne, Clermont-Ferrand, France; 31 Danish Centre for Health Economics, Department of Public Health, University of Southern Denmark, Odense, Denmark; 32 Centre for Telepsychiatry, Region of Southern Denmark, Denmark; 33 Orygen, Parkville, Victoria Australia; 34 Centre for Youth Mental Health, The University of Melbourne, Parkville, Victoria Australia

**Keywords:** Tailored implementation, Normalization, Implementation strategies, Determinants of practice, eHealth, Mental health, Internet-delivered Cognitive Behavioural Therapy, iCBT, Stepped wedge trial design, SWT

## Abstract

**Background:**

Internet-based Cognitive Behavioural Therapy (iCBT) is found effective in treating common mental disorders. However, the use of these interventions in routine care is limited. The international ImpleMentAll study is funded by the European Union’s Horizon 2020 programme. It is concerned with studying and improving methods for implementing evidence-based iCBT services for common mental disorders in routine mental health care. A digitally accessible implementation toolkit (*ItFits-toolkit*) will be introduced to mental health care organizations with the aim to facilitate the ongoing implementation of iCBT services within local contexts. This study investigates the effectiveness of the *ItFits-toolkit* by comparing it to *implementation-as-usual* activities.

**Methods:**

A stepped wedge cluster randomized controlled trial (SWT) design will be applied. Over a trial period of 30 months, the *ItFits-toolkit* will be introduced sequentially in twelve routine mental health care organizations in primary and specialist care across nine countries in Europe and Australia. Repeated measures are applied to assess change over time in the outcome variables. The effectiveness of the *ItFits-toolkit* will be assessed in terms of the degree of normalization of the use of the iCBT services. Several exploratory outcomes including uptake of the iCBT services will be measured to feed the interpretation of the primary outcome. Data will be collected via a centralized data collection system and analysed using generalized linear mixed modelling. A qualitative process evaluation of routine implementation activities and the use of the *ItFits-toolkit* will be conducted within this study.

**Discussion:**

The ImpleMentAll study is a large-scale international research project designed to study the effectiveness of tailored implementation. Using a SWT design that allows to examine change over time, this study will investigate the effect of tailored implementation on the normalization of the use of iCBT services and their uptake. It will provide a better understanding of the process and methods of tailoring implementation strategies. If found effective, the *ItFits-toolkit* will be made accessible for mental health care service providers, to help them overcome their context-specific implementation challenges.

**Trial registration:**

ClinicalTrials.gov NCT03652883. Retrospectively registered on 29 August 2018

## Background

Common mental health disorders account for a large proportion of the global burden of disease [1]. Ample studies report on the clinical impact and other advantages of digital treatment for multiple mental disorders such as depression and anxiety, settings such as primary care or specialized care, and patient groups differing in diagnosis, severity levels, and comorbidities (e.g. [1–4]). Most frequently investigated are Internet-delivered Cognitive Behavioural Therapy (iCBT) services. Recent meta-analyses showed that self-guided iCBT is beneficial for patients with depressive symptoms [5, 6], and guided iCBT was found to have equivalent overall effects compared to face-to-face therapy for the treatment of psychiatric and somatic disorders [7]. Currently, research focusses on developing integrated treatment protocols blending face-to-face therapy with online treatment modules [8].

It is well recognized that evidence alone does not guarantee the effective use of an intervention in routine daily health care practice [9]. The implementation of iCBT services in routine care has varying degrees of success. Implementation is an intentional and planned process of normalizing (i.e. integrating and embedding) an innovation within an organization [10–12]. This process takes place at multiple organizational levels involving a wide range of stakeholders such as health care professionals, managerial staff, and/or patients. Cognitions and behaviours of clinicians and patients as well as organizational procedures are likely to remain in habitual patterns due to complex settings and working mechanisms [13]. Poor implementation contributes to the currently limited uptake numbers of evidence-based psychological treatments such as iCBT services in practice [14–19]. Few scientific studies have been published which systematically investigate and test implementation strategies and plans of such interventions. Efforts in the field primarily focussed on identifying and categorizing the factors hindering or enabling implementation processes. Folker and colleagues [20] described the scepticism of therapists and managers towards the use of iCBT, difficulties with the stable recruitment of patients, and problems with ensuring the long-term sustainability of the iCBT service. Other studies reported the general motivation and belief of professionals regarding the benefits of iCBT treatments [21, 22], but showed that lack of time, inadequate knowledge of the service, and the need to change habits were an impediment to the uptake of iCBT [22]. This is confirmed by a systematic review summarizing the determinants of practice regarding the implementation of Internet-delivered treatments [23]. Determinants of practice refer to any factor that hinders, enables, or facilitates the implementation process. The review highlighted 37 determinants to implementation on health care system, organizational, professional, and patient level showing that there is a multitude of barriers to overcome in order to implement iCBT successfully in routine practice. Depending on the context in which the implementation takes place and the nature of the service to be implemented, barriers differ in number and magnitude and might change over time. In that sense, every implementation setting is unique [24].

### Tailored implementation

In order to overcome local barriers to implementation, suitable implementation strategies need to be applied. Implementation strategies refer to any kind of action aimed at accomplishing the integration of the innovation in practice (e.g. [18, 24–26]). Advances in the field of implementation science explore *tailored* approaches to develop implementation strategies. Tailored implementation strategies are defined as ‘strategies to improve professional practice that are planned, taking account of prospectively identified determinants of practice’ [27]. A Cochrane review of 32 studies showed that tailored implementation can be effective in comparison with non-tailored implementation or no implementation interventions with small to moderate effect sizes when implementing practice guidelines in various health care settings [27]. They highlighted the importance of developing suitable methods to identify local determinants and to subsequently pair them to matching strategies. Following on from this work, the literature suggests that structured group methods involving stakeholders, such as brainstorming and focus groups, are successful methods to identify locally relevant determinants of practice [28]. A focus should be on the local contexts where the determinants emerge, as well as on the prioritization of certain determinants over others [28]. Besides differences in the type of innovation, technical infrastructures, and organizational processes, local contexts might also differ in their implementation culture and leadership. Organizational climates can be conductive to implementing evidence-based interventions, and leaders can employ various strategies to motivate and inspire others to implement innovative practices [29].

**Fig. 1:**
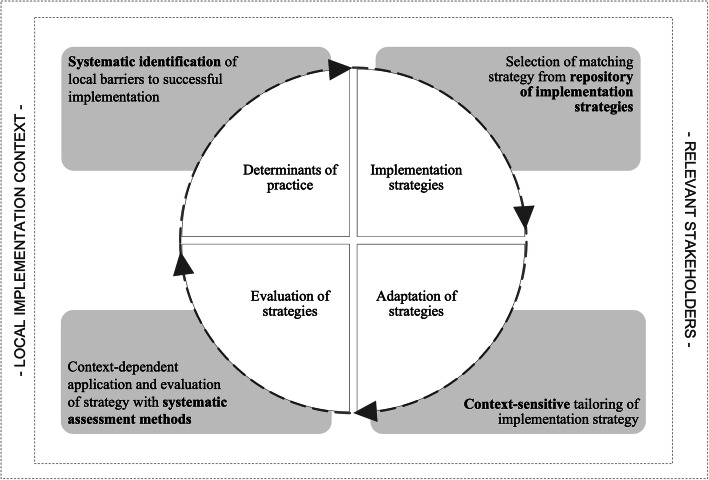
Process of context-specific tailoring as applied in the ImpleMentAll project

Building on previous research [27, 28, 30], the ImpleMentAll project defines the concept of tailored implementation as follows: a prospective process involving systematic identification of *determinants of practices* within a local context, selection of *implementation strategies* appropriate to those determinants, the integration of these strategies into local work structures, and the actual application, evaluation, and potential further adaptation of the tailored implementation strategies (Fig. 1). Thereby, tailoring is assumed to be a universal process applicable across health care contexts, settings, and care disciplines. In order to identify the most relevant determinants and the most suitable strategies to a local context, the identification process should be conducted using systematic methods [31]. The process of identification needs to involve a diverse group of stakeholder opinions to identify a variety of obstacles deemed important to the local situation [27]. As these considerations may substantially vary over time due to changes in internal and/or external circumstances of the organization, continuous tailoring throughout the implementation processes is of importance. Full details of the project-specific conceptualization of tailoring and its rationale will be discussed in a forthcoming publication.

### Objectives

ImpleMentAll aims to evaluate the effectiveness of tailored implementation in integrating and embedding evidence-based iCBT services in routine mental health care for common mental disorders in adults. The projects conceptualization of tailored implementation is operationalized in an online platform, the *ItFits-toolkit*. Health care organizations, including primary and specialist mental health care, currently implement various types of iCBT services around the globe [32]. The ImpleMentAll consortium will use this natural laboratory to test whether the *ItFits-toolkit* will lead to favourable implementation outcomes compared to *implementation-as-usual* in twelve ongoing implementation initiatives of iCBT in routine care in nine countries in Europe and Australia. Successful implementation is hereby—primarily—defined as the normalization of the use of the iCBT services within the local settings. This paper presents the ImpleMentAll study protocol. The reporting follows the CONSORT extension for stepped wedge cluster randomized trials [33].

## Methods

### Trial design

**Fig. 2:**
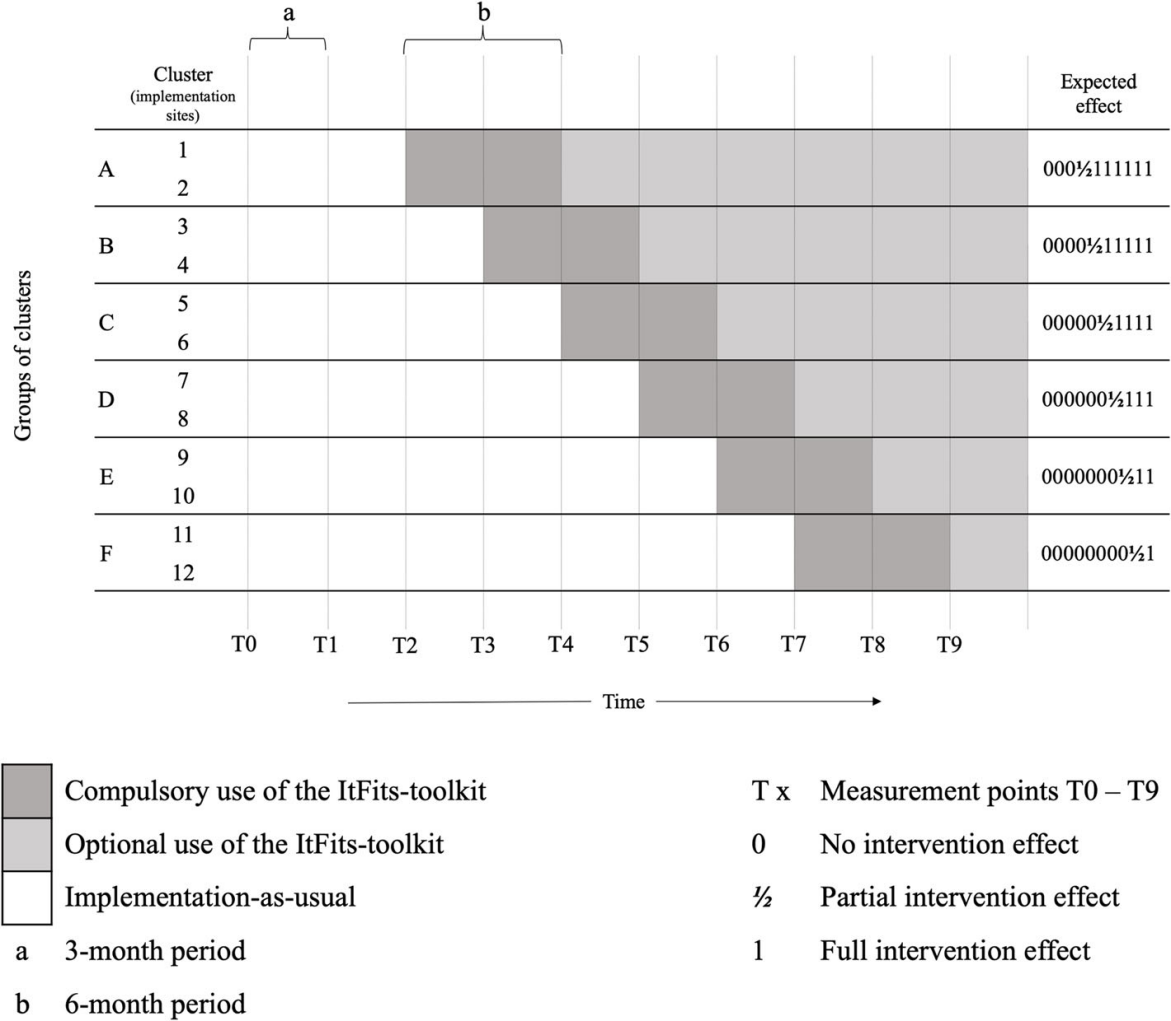
Stepped wedge cluster randomized trial design for the ImpleMentAll project

A closed cohort stepped wedge cluster randomized controlled trial (SWT) design will be applied. Figure 2 schematically represents this design. Within a time period of 30 months, the *ItFits-toolkit* will be rolled out sequentially to twelve implementation sites (clusters). The twelve clusters are randomly allocated to six sequences (A–F) defining the different time points at which the clusters will cross over from the control condition (*implementation-as-usual*) to the experimental condition (*ItFits-toolkit*). As such, each cluster acts as both the control and experimental group over time. The successive cross-over points are scheduled every 3 months. The *ItFits-toolkit* will be used by each cluster for a minimum of 6 months (*b* = minimal exposure period). During the 6-month exposure time, sites will receive technical support. The cohort will be encouraged to continue using the *ItFits-toolkit* after the minimal exposure period. Due to a potential intervention lag effect, it is expected that changes in the outcome measures become gradually visible in the data within and after the 6-month exposure period. As such, the effect is hypothesized to increase from no effect in the control condition (0) to a partial effect during the compulsory exposure period (½) to a full and lasting effect after the 6-month exposure (1). Data will be collected 3-monthly (T0–T9) to strike a balance between the ability of the measurements capturing change over time and the measurement burden. A pre-rollout period of 6 months is chosen to obtain stable measures of implementation-as-usual activities in all clusters. That means the first three measurements (T0–T2) consist solely of *implementation-as-usual* data and at T2 the first two clusters cross over to the experimental condition, followed by two clusters every 3 months.

### Study setting

Twelve implementation sites from nine countries—Italy, Spain, Germany, France, The Netherlands, Denmark, Kosovo, Albania, and Australia—form the natural health care laboratory for this study. The health care systems across the implementation sites differ regarding the organization of mental health service delivery, including aim and type of iCBT services offered (treatment or prevention, and self-help, guided, or blended format), clinical pathways, guidelines, procedures, and cultures, as well as financing and legislative. Within those care settings, the participating implementation sites’ mental health services are located in community care, in primary or specialized care, or in a stepped-care model. Referral pathways include self-referral, as well as referral by GPs, psychologists, psychotherapists, or insurance companies.

All implementation sites have adopted and are implementing prevention or treatment services of mild to moderate depressive disorder, anxiety disorder, substance abuse, and medically unexplained symptoms or somatic symptom disorders. The iCBT services are based on the working mechanisms of Cognitive Behavioural Therapy covering four main components: psycho-education, techniques invoking behavioural change, a cognitive component, and relapse prevention [34]. All services make use of Internet technology. However, the specific operationalization differs per service in response to the local requirements. Similarly, various guidance modalities are embedded in the iCBT services, ranging from self-help with minimal technological and administrative support, to therapist guided treatments, and blended approaches where online modules and face-to-face therapy are integrated into one treatment protocol. Patient pathways, clinical eligibility criteria for receiving the iCBT service, as well as stopping rules of participation follow local guidelines and procedures applicable in the implementation sites.

### Participants

**Fig. 3:**
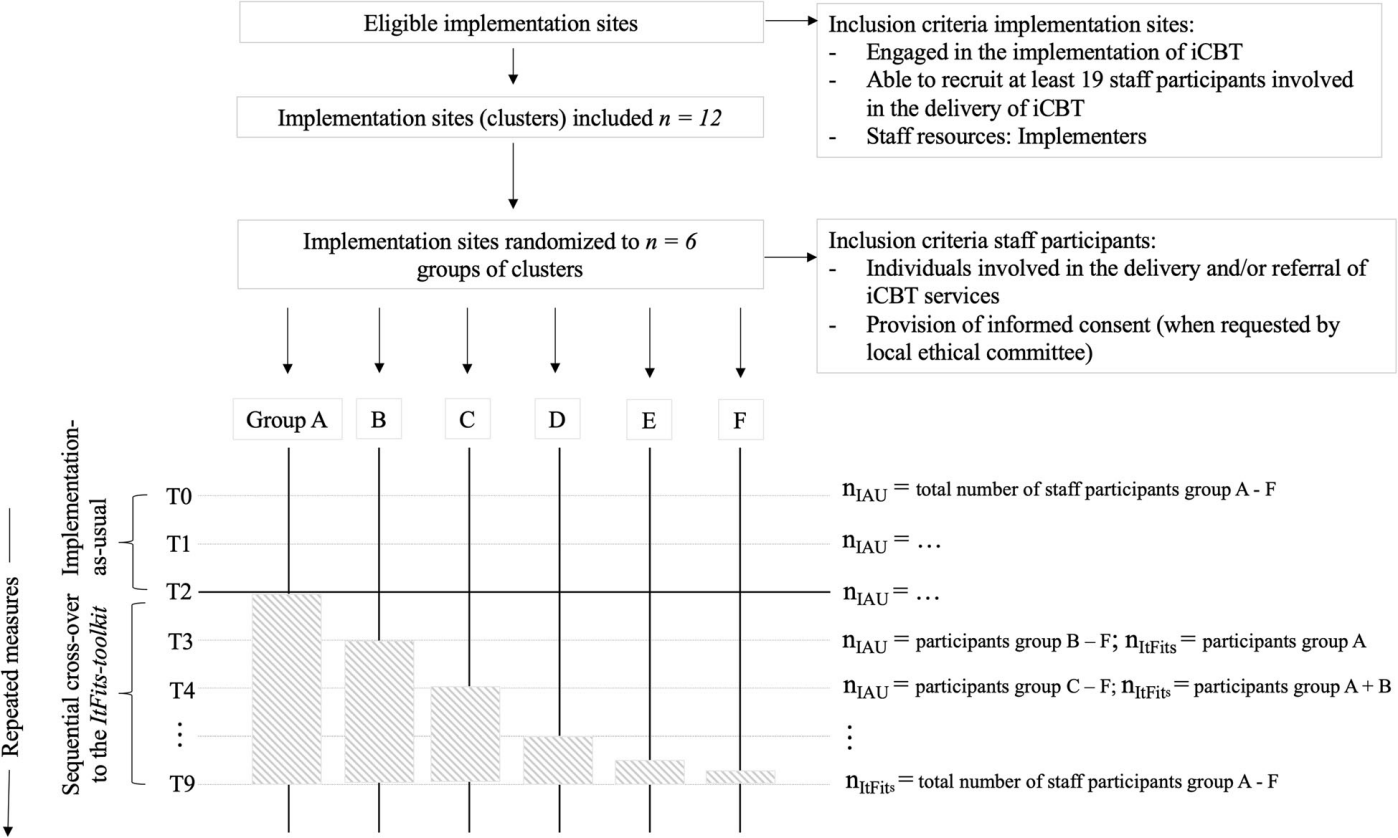
Anticipated participant flow. The total number of participants will be calculated by summing up participants across all groups and all measurement waves

Following the SWT design reporting guidelines [33], participants are classified at two levels: (1) implementation sites (organizations) as cluster-level participants represented by individuals responsible for the local implementation work (implementers) and (2) staff within these sites as individual-level participants. The anticipated participant flow through the study is schematically depicted in Fig. 3.

#### Organizational (cluster) level

The implementation sites eligible to partake in the study are engaged in the implementation of iCBT at least 3 months prior to the baseline measurement. Each implementation site is responsible to recruit a sufficient number of staff participants (see the “Sample size and power estimates” section).

#### Staff (individual) level

Every individual involved in the delivery of the iCBT service within the participating implementation site is eligible to take part in the study. Staff participants can have different roles in the delivery of iCBT and include therapists, such as psychologists, psychiatrists, or mental health nurses; referrers such as GPs, pharmacists, community workers, health promotors, counselors, or case managers; administrators such as clerical workers or secretaries; ICT support staff, such as security officers, maintenance officers, or helpdesk staff; and managers of the organizations. Staff participants will give their informed consent in accordance with local and European directives for the protection of privacy, voluntary participation, and the right to withdraw from the study at any given time. Staff is excluded from participation when they are enrolled in the study as local implementers or when they are involved in any activities of the local or central trial management.

### Conditions

#### The experimental condition: the *ItFits-toolkit*

The online self-help implementation toolkit ‘*ItFits*’ aims at supporting implementers in developing, applying, and monitoring implementation strategies that are adapted to local contexts to integrate and embed iCBT services in routine mental health care. The *ItFits-toolkit* has the potential to impact the implementation on various levels (e.g. at staff, patient, organizational, and policy level). Examples may include the adaptation of organizational workflows, personnel decisions, training and motivation, or modifications of the service delivery mode. The *ItFits-toolkit* is based on scientific output and theories in the field of implementation [30, 35, 36]. To ensure an appropriate balance of being theoretically informed whilst also practically orientated and accessible to non-academic users, the *ItFits-toolkit* has undergone rounds of conceptual and technical piloting, with user groups representing a range of relevant perspectives.

**Fig. 4:**
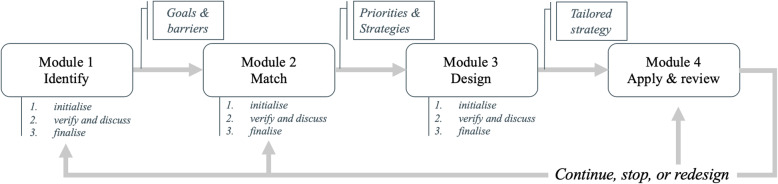
Flowchart of the *ItFits-toolkit*

Within each implementation site in the ImpleMentAll study, a self-guided implementation core team (up to four staff members internally to the organization (implementers) represented by an implementation lead) will be established. These teams are likely to include therapists and other professionals involved in the delivery of the iCBT service, but may also include individuals from partner organizations where appropriate, for example, if they are invested stakeholders in the service (e.g. commissioners). The implementation core team will coordinate and work with the *ItFits-toolkit*. In four modules, concrete guidance on tailoring implementation strategies to local determinants of practices will be provided, applied, and evaluated. The four modules are (1) identifying and prioritizing implementation goals and determinants of practices, (2) matching up implementation determinants to strategies, (3) designing a plan for carrying out strategies in a local context, and (4) applying strategies and reviewing progress. In the last module, a decision will be made whether the implementation strategy will be stopped (in case of perceived success), continued, or redesigned. Figure 4 illustrates the workflow of the *ItFits-toolkit*. An overview of the main working components of the *ItFits-toolkit* is summarized in Table 1.

**Table 1:** Core working principles of the *ItFits-toolkit*

1) Flexible, systematic step-by-step workflow	
2) Stakeholder-based co-creation to reach consensus	
3) Tools to identify local barriers, consult stakeholders, and match to suitable strategies	
4) Evidence-informed materials on barriers, strategies, and intervention planning	

**Fig. 5:**
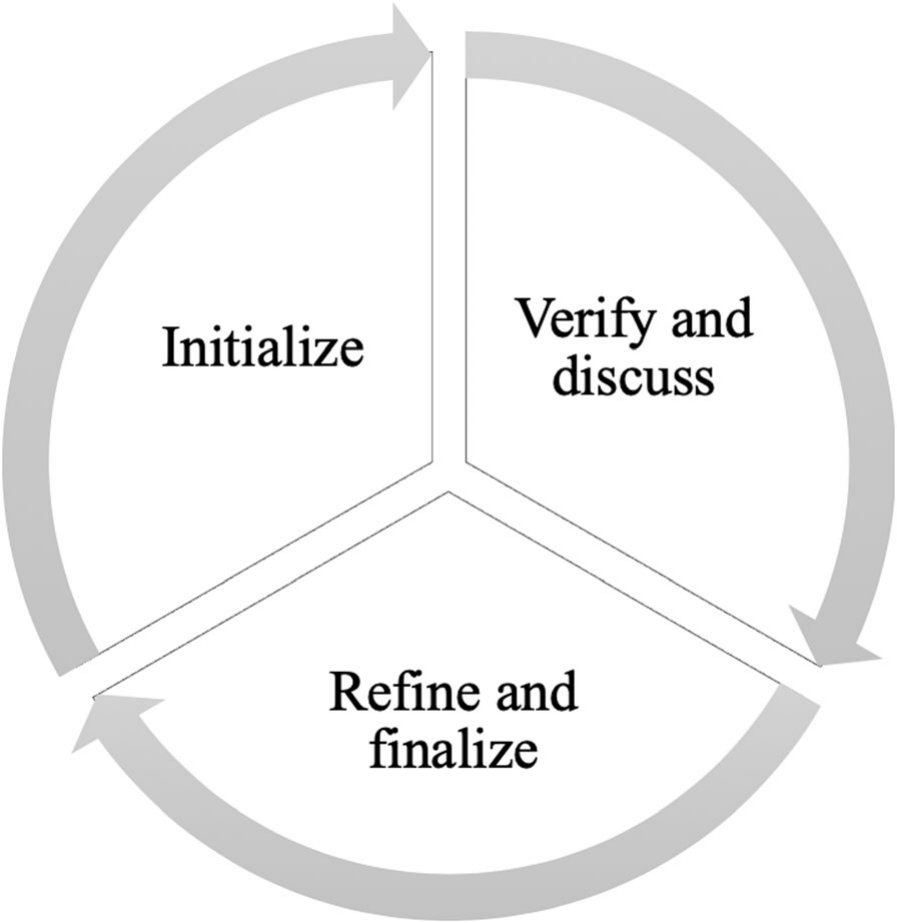
Three-step iterative working process for each module to reach consensus among relevant stakeholders

Within the four modules, the *ItFits-toolkit* employs a systematically guided but flexible step-by-step process, including stakeholder-based co-creation. The Normalization Process Theory (NPT) explains that successful integration and embedding are achieved through people (individuals and groups) working together [35]. Therefore, engagement and consultation of staff involved in the implementation work and in iCBT service delivery are a core feature of the toolkit. Stakeholders are individuals which either actively affect the iCBT service (e.g. service delivery staff, IT staff, managers) or are passively affected by the delivery of the iCBT service (e.g. patients). Implementers work through a three-step iterative process (see Fig. 5) in order to reach the best possible outcome for each module. The local implementation core team develops an initial plan or idea, which is discussed with and reviewed by the stakeholders for feedback in order to design a feasible plan that reflects the needs, priorities, and restraints in the local situation. Subsequently, the implementation core team finalizes the plan to accommodate stakeholders’ feedback. For each module, different stakeholders might be consulted depending on the task at hand.

In order to engage with stakeholders throughout the process, a number of consensus techniques are recommended, including brainstorming, structured group discussions, individual and informal discussions, and surveying [28]. Surveying functionalities are embedded within the *ItFits-toolkit* to allow implementers to flexibly create dedicated online surveys which they can administer to relevant stakeholders via email. In addition, the toolkit allows implementers to upload and store notes, audio recordings, and other relevant materials, which document the decisions and progress made and can be used for reviewing purposes. Implementers are also actively working with evidence-informed materials, including literature on iCBT relevant determinants of practice [23, 37, 38] and implementation strategies [25, 26, 39], as well as guidelines to develop and structure the tailored implementation plan [40].

The commitment of study sites to participate in the trial and to use the toolkit according to the study protocol has been agreed in advance, and resources have been allocated to support their participation. Each participating site is required to work with the toolkit for at least 6 months and is instructed to strive to finish all four modules within this time period. The intensity of use depends on the local context and needs. For the toolkit, a protocolized introductory training will be provided, and periodic support (in form of monthly support sessions and assistance on request) will be available, focusing solely on technical questions, such as login procedures, the use of tools, or the navigation through the platform, to ensure a smooth working process without interfering with the working components of the toolkit. Within the toolkit itself, there is access to written, audio, and video guidance to completing activities within the modules. The training and support will be provided by two members of the research team.

#### The control condition: *implementation-as-usual*

*Implementation-as-usual* functions as the control condition in testing the effectiveness of the *ItFits-toolkit*. It refers to any existing activities the implementation sites are engaged in to embed and integrate the local iCBT programme within routine care. Examples include the provision of training to iCBT service deliverers, educating staff and patients on the use of iCBT, or media campaigns to raise awareness of iCBT services. The implementation sites started the processes of routinely implementing the iCBT services at least 3 months prior to baseline (T0) with the goal of continuously improving the utilization of the services.

### Outcomes

#### Primary outcome

The effectiveness of the *ItFits-toolkit* will be expressed in the extent to which the *ItFits-toolkit* is able to increase the degree of normalization of the use of the iCBT services compared to usual implementation activities. The degree of normalization is the extent to which staff involved in service delivery and referral consider the iCBT service to be a normal part of their routine practice. It will be measured with the 20-item Normalization MeAsurement Development tool, short NoMAD [41, 42]. The NoMAD is a self-report instrument measuring the degree of normalization by focusing on four constructs as defined by the Normalization Process Theory [35]: coherence, cognitive participation, collective action, and reflexive monitoring. The NoMAD shows high internal consistency and has been validated in heterogeneous samples across languages and settings [42–44].

#### Exploratory outcomes

##### Service uptake

Service uptake is defined in terms of the completion rate of the iCBT service, that is, the absolute number of patients actually completing the trajectory of the iCBT service during the trial period. Each implementation site has their own protocolized or experience-based operationalization of ‘completion’ according to the therapeutic principles and procedures of the local service being implemented. In addition to collecting data on completion rates, also data on referral rates will be collected to investigate the effect of the *ItFits-toolkit* on iCBT service uptake by staff, i.e. referral to the service.

##### Implementation costs

Efficiency is defined as the ratios between implementation cost and service uptake, and implementation cost and degree of normalization. Within the ImpleMentAll study, implementation costs are defined as the sum of personnel costs, other direct costs, and indirect costs. Personnel costs are calculated by multiplying the working hours spent by the implementers on implementing the iCBT service by standardized hourly wage rates and a standardized country correction factor. Other direct costs include costs for consumables, equipment, and services purchased for the purpose of carrying out the implementation activities. Indirect costs are costs that cannot be attributed directly to the implementation work but are necessary to facilitate the implementation, such as office rent, office IT infrastructure, or administration. Indirect costs are calculated by taking 20% of the direct costs. Implementation costs (i.e. personnel costs, other direct costs, and indirect costs) will be assessed in the local implementation settings by monitoring the different cost components over time.

##### Exposure

Exposure to the *ItFits-toolkit* will serve as a measure to determine if the measured change in outcomes can reliably be related to the use of the toolkit. Usage data will be automatically collected by system logs recording time stamped starting and stopping of *ItFits*-modules (use) and a binary confirmation of the existence of output of the modules (result).

##### Satisfaction

As the *ItFits-toolkit* is a newly developed tool*,* satisfaction of use will be assessed to establish to what extent the toolkit is able to fulfil implementers’ needs and expectations in tailored implementation. Satisfaction will be assessed with the short version of the Client Satisfaction Questionnaire (CSQ-3) [45], which has good psychometric properties and has been tested in numerous studies and diverse samples [46, 47].

##### Usability

Usability will be measured with the validated System Usability Scale (SUS) [48, 49]. The instrument measures the perceived, local usability of the *ItFits-toolkit*—such as complexity of the tool, user experience, and perception of the technological realization—by the toolkit user.

##### Impact

The perceived impact will be assessed to explore whether the implementation strategies developed by using the *ItFits-toolkit* are considered to be satisfactory in fulfilling the implementers’ needs. The perceived impact will be measured using a visual analogue scale.

##### Organizational readiness

Organizational readiness for implementing change [50, 51] is hypothesized to be a potential precursor or contextual factor for implementation success [52]. This concept will be assessed using the ‘Organizational Readiness for Implementing Change’ (ORIC) questionnaire [51], which focuses on the psychological and behavioural preparedness of members of an organization to implement organizational change [51]. Considering similarities in theoretical concepts, ORIC questionnaire data will also be used to explore its convergent validity with the NoMAD instrument.

Table 2 summarizes the primary and exploratory outcomes. All staff-level questionnaires have been translated and adapted into local languages using a standardized translation guide. The translated versions of the instruments are available for public use [53].

**Table 2:** Primary and exploratory outcomes of the ImpleMentAll study

	Outcome	Instrument	Organizational level	Staff level
*Primary outcome*	*Degree of normalization*	NoMAD (20 items)	–	Baseline, 3-monthly
*Exploratory outcome*	*Demographics*	Self-developed questionnaire	Once during the study period	Baseline
	*Service uptake*	Self-developed questionnaire Data source: administrative data basis (e.g. iCBT platform)	Baseline, 3-monthly	–
	*Implementation costs*	Self-developed questionnaire Data source: financial administration	Baseline, 3-monthly	–
	*Exposure*	Event-based platform log-files	Continuous	–
	*Satisfaction*	CSQ (3 items)	End of exposure time	–
	*Usability*	SUS (10 items)	End of exposure time	–
	*Perceived impact*	Visual analogue scale (1 item)	End of exposure time	–
	*Organizational Readiness*	ORIC (12 items)	–	Baseline, 3-monthly

Outcomes, assessment instruments, the level on which the outcomes are assessed, and measurements’ time intervals. All staff-level questionnaires have been translated and adapted into local languages using a standardized translation guide

### Data collection

Data will be collected on implementation sites and staff level through (1) a central data management system specifically built for the purpose of the study and (2) event-based log files of the *ItFits-toolkit*. Online surveys will automatically be sent to participants via email with the request to fill in a specified measure. The measurement time points are pre-scheduled for 3-month intervals during the 30-month study period. Data on demographics are collected once, when a participant enters the study. Degree of normalization, uptake, implementation costs, and organizational readiness for change will be measured at 3-monthly intervals. Exposure data will be collected continuously, and data on satisfaction and usability will be recorded at the end of the exposure period (see Table 2). Depending on the participant’s activity, reminders will be sent at regular intervals to ensure continuous completion of the questionnaires by each participant. The burden for study participants to provide the required data is kept to a minimum by using brief online questionnaires and automatically collected data.

### Sample size and power estimates

This study has a fixed cluster sample size by design (*n* = 12 implementation sites) based on availability and willingness of organizations engaged in implementation of iCBT services to conform to the study’s eligibility criteria. For the staff-level outcome, a series of simulation studies was conducted using a multi-level degree of normalization data (i.e. NoMAD items) to estimate the minimal required number of staff members to sufficiently power the analysis. Here, a 5% increase in absolute normalization scores and an increased 3-month growth rate from .05 to .10 are assumed to be statistically decisive in superiority for either condition. The cluster sample size of 12 clusters, with 15 staff participants per implementation site per measurement wave, achieves > 80% power to detect this effect, using a two-sided test with a significance level *α* of .05. Taking a conservative study drop-out of 20% into account, the minimum staff sample size was set to *n* = 19 per implementation site. In line with the closed cohort design, each participant will be measured continuously over a period of 10 measurement waves. For all 12 clusters, this results in a total minimum sample size of 228 staff participants with 2280 repeated data points.

### Randomization

Implementation sites will be randomly allocated to one of six groups (two implementation sites per group, see Fig. 3) prior to the start of the study. Randomization will be conducted by a computerized random number generator using R [54]. No constraints to randomization will be applied. The allocation scheme can only be accessed by the central trial coordination team. Any other investigators and all study participants within the implementation sites will be blinded to the crossover sequence. Three months prior to cross-over, the two clusters randomized for rollout, the process evaluation team and the team involved in supportive activities of these sites, will be informed in order to prepare any organizational prerequisites necessary for using the *ItFits-toolkit*.

### Statistical methods

For the normalization outcomes, a three-level GLMM will be conducted, with ‘Wave’ clustered at the ‘Staff’ level, and ‘Staff’ clustered at the ‘Site’ level, accounting for the correlation structure in the outcome. Random effects will be used to assess correlations between observations within and across units in the same clusters. Each regression parameter, including the intercept, will be allowed to vary within cluster levels ‘Staff’ and ‘Site’. Efficiency of the implementation process (i.e. normalization and service uptake divided by implementation costs) will be included in the separate analyses.

It will be tested whether the introduction of the *ItFits-toolkit* influences iCBT service uptake by patients across and within sites [55, 56]. An effect of the toolkit is demonstrated when service uptake shows a significant main effect of *ItFits-toolkit* use or an interaction effect of *ItFits-toolkit* use and measurement wave (time). Consequently, trial data will be analysed using generalized linear mixed modelling (GLMM) [55] with service uptake as the dependent variable, and measurement wave (time), *ItFits-toolkit* use (yes/no), and interaction between time and *ItFits-toolkit* use as independent variables. To account for the expected intervention lag effect, a fractional term for the ‘*ItFits*’ parameter will be included in the 6-month minimal exposure time (ranging from 0 to 1, i.e. 0–½–1). Service uptake outcomes will be modelled in a two-level GLMM, since these measures are collected at the site level only. Thus, to account for the correlation structure of the uptake outcome, ‘Wave’ is modelled to be clustered at the ‘Site’ level. All regression parameters will be allowed to vary.

For exploratory purposes, measures of exposure to the *ItFits-toolkit* (event-based log files showing intensity of use and level of continuous use), CSQ, SUS, and ORIC questionnaire data will be added as additional predictors of outcome in the above-described regression models.

In the analyses, all observed data will be included following the intention-to-treat principle. The ability of mixed models to estimate model parameters in the presence of missing observations will be used, and increased uncertainty caused by missing values will be accepted as a given quality of the results.

### Process evaluation

#### Implementation-as-usual

The *implementation-as-usual* process evaluation will explore *implementation-as-usual* activities in which implementation sites were engaged in prior to receiving the *ItFits-toolkit*. This analysis will identify and describe these implementation actions and determinants they focused on.

#### The *ItFits-toolkit*

A qualitative process evaluation will be conducted to study how the effects of the *ItFits-toolkit* were achieved and to obtain a better understanding of the underlying theoretical and conceptual mechanisms of tailored implementation. The process evaluation will focus on (1) understanding what implementers do with the *ItFits-toolkit*, (2) understanding and describing how the *ItFits-toolkit* gets reconfigured and adapted within and across settings when it is used, and (3) identifying, describing, and understanding the micro-, meso-, and macro-mechanisms that shape *ItFits-toolkit* use within and between implementation sites. The *ItFits-toolkit* process evaluation will be theoretically informed by Normalization Process Theory (NPT) [35], Self-determination Theory (SDT) [57], and work within organization studies, especially on organizational routines [58, 59]. The conceptual ideas within these bodies of literature will enable the research team to focus on the work through which the *ItFits-toolkit* is implemented, what motivates individuals to work with the *ItFits-toolkit*, and how the *ItFits-toolkit* facilitates the structuring of time, resources, and people to support implementation. Four qualitative research methods will be used within the process evaluation, including theory-informed interviews with Implementation Leads and main informants (e.g. implementation practitioners and trainers of the *ItFits-toolkit*), in situ and distal observations of implementers engaged with the *ItFits-toolkit*, process data from the use of the *ItFits-toolkit*, and analysis of documents, texts, and technological specifications produced and made available by *ItFits-toolkit* users. Interviews with the Implementation Leads will be generally conducted in English. More focused qualitative observations will be conducted in some purposively sampled sites according to the spoken languages of the researchers. This work will involve in situ and distil observations of meetings of the core implementation teams and meetings with key stakeholders. Qualitative data will be analysed according to the analytical framework developed and will be conducted according to the standard procedures of rigorous qualitative analysis [60]. Analysis will occur concurrently with data collection following the stepped order of implementation sites’ entry into the trial. This allows for emerging trends found in earlier rounds of fieldwork to be explored in subsequent ones. All data will be audio-recorded, transcribed verbatim, and analysed using framework analysis [61]. The results of the *ItFits-toolkit* process evaluation will be used to further inform the outcome evaluation.

## Discussion

The ImpleMentAll study is a large-scale international collaborative research project designed to study the effectiveness of tailored implementation and better understand the mechanisms of implementing iCBT for common mental disorders. A newly developed digitally accessible toolkit by which implementation strategies are prospectively developed, adapted, applied, and evaluated will be tested for its effectiveness compared to usual implementation activities. The toolkit will be introduced in twelve different mental health care organizations in nine countries across Europe and Australia. This real-world research setting provides a variety in health care systems, iCBT services, policies, implementation climate, and levels of experience in delivering iCBT. Tailored implementation is thought to be generically applicable across care contexts. The conceptual idea behind the tailoring process builds on recent literature findings and methods in the field of implementation (publication forthcoming).

The ImpleMentAll project applies a stepped wedge cluster randomized controlled trial design to determine the effectiveness of the *ItFits-toolkit*. Reasons for choosing the SWT design include practical feasibility and flexibility, fairness, and strength of the evidence [56]. A classical randomized controlled trial would not have been feasible due to the highly heterogeneous and reasonably small sample of organizations included in the study. It would not have been possible to alternatively randomize participants at individual level as this would have conflicted the naturalistic setting of the study. Due to repeated measures, a SWT requires less participants to adequately power the statistical analysis. However, achieving and maintaining a stable sample for the duration of the study will be challenging. Biases due to time trends such as organizational restructuring, data regulation policies and legislation, and technological advances need to be considered given the potential large intervention lag effect in implementation trials. A SWT design allows for the possibility to adjust for time trends in outcomes. It distributes the chances of such time trends affecting the dependent variables equally across the participating sites. Furthermore, the design increases practical feasibility of the study as it allows for sequential, batch-wise training of the implementation sites to the *ItFits-toolkit* and keeps guidance limited to those groups who are in the exposure condition.

The use of such a trial design is novel, but the literature in the field is growing at the time of writing. Generic guidance [33, 62] on how to conduct a SWT is available. However, there is no scientific literature on particular components of the design applied to implementation research, most notably the minimal exposure time to exert an effect in relation to the potential intervention lag effect. A minimal exposure period of 6 months was chosen as it strikes a reasonable balance between time constraints of the total trial period and to constitute to meaningful exposure by finishing one complete cycle of the *ItFits-toolkit*. Carry-over effects might occur when implementation sites cross over from the control to the experimental condition. The process evaluation conducted within this project is expected to shed light on the implementation mechanisms considering potential carry-over effects. This will be regarded when interpreting the results of the effectiveness study.

The ImpleMentAll study does not include an evaluation of the clinical effectiveness of the iCBT services per se, as the focus lies on establishing the effectiveness of the *ItFits-toolkit*—the implementation intervention*.* The effectiveness of the *ItFits-toolkit* will be investigated by measuring implementation outcomes mainly on organization and staff level, anticipating that change processes to successfully implement iCBT in routine care are predominantly taking place at those levels. The perspective of the patient using the iCBT services will be considered indirectly by assessing the completion rates of the services by the patients. This means that the implementation could be perceived as successful from the perspective of the organization and staff, irrespective of improvements in clinical outcomes (e.g. symptom reduction) in the patient.

The field of implementation research is still young, and therefore, access to thoroughly validated and theory-based measurement instruments is limited [63, 64]. The measurement instruments used to assess implementation success or related outcomes (i.e. NoMAD, ORIC, and implementation costs measure) are relatively new, though promising [42–44, 51]. Experience of the field to use these validated measures is low, and therefore, the interpretation might not be as straightforward compared to well-established instruments. Uncertainties around sensitivity to change in these instruments are present, and further psychometric validation is planned.

The ImpleMentAll study engages in a number of challenges, mostly related to the relatively new concepts under study, the fast-changing world of technology-assisted interventions, and the complex and heterogenous implementation contexts. In that sense, the ImpleMentAll study is well-positioned to take the first step towards exploring the effectiveness of an online self-help toolkit for a tailored implementation supporting the implementation of evidence-based eHealth in mental health care. As such, it will contribute to implementation science by investigating the effectiveness of tailored implementation and providing a better understanding of the process and methods for tailoring implementation strategies. Measurement instruments for implementation outcomes related to implementation success will be further improved and validated. If effective, the *ItFits-toolkit* will be made available to implementers supporting them in identifying barriers, selecting, localizing, and applying appropriate implementation strategies for successfully implementing iCBT in their practices. This will ultimately be beneficial for the large proportion of individuals in need of evidence-based health care.

### Trial registration

This protocol was registered with ClinicalTrials.gov on August 29, 2018 (No. NCT03652883). Results of the study will be reported to ClinicalTrials.gov. The research team will monitor protocol compliance and record the progress of the study. The principal investigator will submit annual reports on study progress to the European Commission, the main funder of the project.

## Trial status

The ImpleMentAll study runs from March 2018 to March 2021. Implementation sites across Europe and Australia were recruited following a purposeful sampling approach. According to the closed cohort design of the study, recruitment of clusters (implementation sites) was completed prior to the first measurement wave (before September 2018). Subsequently, the included implementation sites engaged in recruiting a minimum number of staff participants (*n* = 19 per cluster) to ensure a stable and sufficient sample for repeated data collection during the trial. Staff participant recruitment is open and continues throughout the trial to allow for replacements of potential study drop-outs. Implementation sites have localized the study protocol, translated measurement instruments and obtained ethical approval. Ethical approval for the process evaluation was granted by the University of Northumbria, UK. The randomization scheme has been finalized. Data collection will be completed in late 2020 and the first results are expected to become available in 2021.

## Data Availability

The translations of the measurement instruments NoMAD and ORIC are available for public use and can be accessed here https://www.implementall.eu/9-outcomes-and-resources.html.

## Abbreviations

CBT: Cognitive Behavioural Therapy
CSQ: Client Satisfaction Questionnaire
GLMM: Generalized Linear Mixed Modelling
iCBT: Internet-delivered Cognitive Behavioural Therapy
*ItFits-toolkit*: Integrated Theory-based Framework for Implementation Tailoring Strategies
NoMAD: Normalization Measure Development Questionnaire
NPT: Normalization process theory
ORIC: Organizational Readiness for Implementing Change
SDT: Self-determination Theory
SUS: System Usability Scale
SWT: Stepped wedge cluster randomized controlled trial
